# Food labour, consumption hierarchies, and diet decision-making in Sri Lankan households: a qualitative study

**DOI:** 10.1186/s40795-020-00389-w

**Published:** 2020-11-20

**Authors:** J. Renzella, S. Fernando, B. Kalupahana, P. Scarborough, M. Rayner, N. Townsend

**Affiliations:** 1 Centre on Population Approaches for NCD Prevention, Nuffield Department of Population Health, University of Oxford, Oxford, UK; 2 Sri Jayewardenepura General Hospital and Post Graduate Training Centre, Colombo, Sri Lanka; 3 Tianjin Medical University, Tianjin, China; 4 NIHR Biomedical Research Centre at Oxford and Centre on Population Approaches for NCD Prevention, Nuffield Department of Population Health, University of Oxford, Oxford, UK; 5 Department of Health, University of Bath, Bath, UK

**Keywords:** Food, Cooking, Diet, Health promotion, Gender, Adults, Sri Lanka, Qualitative

## Abstract

**Background:**

Sri Lanka faces the double burden of over- and undernutrition. To tackle this dual challenge, double duty interventions that improve the quality of the Sri Lankan diet in line with national dietary guidelines have been suggested. The success of these interventions depends upon an understanding of the context-specific factors that impact their uptake within the population. The purpose of this study was threefold: explore household responsibility for food-related labour; understand food decision-making influences; and investigate consumption hierarchies that might impact the distribution of intervention benefits.

**Methods:**

We conducted face-to-face semi-structured interviews with 93 Sri Lankan adults residing in urban Colombo (*n* = 56), and urban and rural sectors in Kalutara (*n* = 29) and Trincomalee (*n* = 8). Interview data were analysed thematically.

**Results:**

Findings from this study suggest that women in Sri Lanka continue to shoulder the burden of food-related labour disproportionately to men but that this responsibility is not always a proxy for dietary decision-making power. While men are often absent from the kitchen, their role in food purchasing and payment is prominent in many households. Despite these observed gender differences in food labour and provisioning, *“traditional”* age- and gender-based consumption hierarchies with negative nutrition consequences for women and children are not common, indicating that Sri Lankan ‘table culture’ may be changing.

**Conclusion:**

Dietary interventions with the aim of influencing day-to-day practice should be developed with an awareness of who is responsible for, who is able to perform, and who influences targeted behaviours.

**Supplementary information:**

**Supplementary information** accompanies this paper at 10.1186/s40795-020-00389-w.

## Background

Suboptimal diet is the leading risk factor for global morbidity and mortality, and an important preventable risk factor for noncommunicable diseases (NCDs) [1]. As urbanisation, economic development, and income growth lead to predictable shifts in diet, many low- and middle- income countries entering the fourth stage of nutrition transition now face the double burden of malnutrition (DBM) [2–4]. What follows is the pressing and overwhelming task of tackling these coexisting and contrasting nutrition challenges; one that the World Health Organization (WHO) suggests public health policymakers approach as an opportunity to identify and implement integrated solutions [5, 6]. Sri Lanka is one such country whose progression to the fourth stage of nutrition transition has been accompanied by multiple malnutrition burdens [7]. With a substantial portion of the Sri Lankan adult population failing to consume a balanced and varied diet, interventions that improve the quality of the Sri Lankan diet in line with national dietary guidelines have been suggested [8].

The Sri Lankan Food-Based Dietary Guidelines (SLFBDG) aim to provide healthy diet guidance for the general public that can be ‘practically translated into day-to-day practice’ [9]. To support this translation, the Sri Lankan government aims to increase guideline dissemination; conduct awareness campaigns to promote guideline adherence; and train health workers on the SLFBDG recommendations [10]. The success of such high-agency interventions that provide people with healthy diet advice, guidance, and encouragement is heavily mediated by the targeted individuals’ personal resources: time, knowledge, skills, autonomy, and material means [11]. Successful implementation of the SLFBDG recommendations therefore depends on a clearer understanding of who is responsible for, who is able to perform, and who influences ‘day-to-day practice’. In terms of food preparation, for example, it is well documented that Sri Lankan women, be they Sinhalese, Tamil or Moor, disproportionately shoulder the burden of domestic chores and that men do not share the responsibility for cooking in equal measure [12, 13]. Less is known, however, about household responsibility for other food-related chores such as purchasing, payment, and preparation and how the food preferences of family members influence diet decisions. When it comes to consumption, we know that intervention benefits may not be equally distributed within households if intra-household food distribution subscribes to gender and age biases (hereinafter referred to as consumption hierarchies) [14]. Unequal food distribution, where patriarchal consumption hierarches manifest in inadequate nutrient consumption in women compared to men, has been well-documented in many South Asian countries [15]. There is also some evidence of consumption biases that preference adult men over women and children in the allocation of calories among poor urban families in Sri Lanka [16]. However, variations in cultural norms and food practices make it difficult to generalise the findings from these other South Asian countries for Sri Lanka as a whole.

By investigating food skills, knowledge, decisions, and consumption biases in Sri Lankan households, we gain a contextual understanding of how and for whom interventions aimed at promoting healthy eating might work. With the aim of informing the development and successful implementation of such interventions, this paper has the following objectives:

i. explore the heterogeneous nature of household food-roles and responsibilities;
ii. understand food decision-making influences; and
iii. investigate the presence and potential impact of consumption hierarchies.

## Methods

### Data collection

Between December 2018 and February 2019, we invited one adult from 94 Sri Lankan households in urban Colombo (*n* = 56), and urban and rural sectors in Kalutara (*n* = 29) and Trincomalee (*n* = 9) to participate in interviews. Both Colombo and Kalutara are majority Sinhalese and Buddhist Western provinces whilst the less densely populated Trincomalee district is located in the majority Moor and Muslim Eastern Province. Divisional Secretaries of Colombo, Kalutara, and Trincomalee were contacted to obtain electoral lists for each Grama Niladhari Division (GND) within the district and gain permission to visit individual households for data collection. Adults aged 18 years or older residing in these districts were eligible for study inclusion. Data collection started at a randomly selected location within the district. From that starting point, the nearest house appearing in the relevant electoral list was approached. If a consenting adult was present, the third house to the left was approached for the next interview, and so on. If more than one eligible adult was present in the household, the interviewee was selected by drawing lots. If an eligible adult was not present or did not consent, the house next door was approached. This recruitment method was followed until 56, 29, and nine participants in Colombo, Kalutara, and Trincomalee, respectively, were recruited. Interviews took place as part of a broader quantitative study of a new food recall questionnaire which was powered to detect similarities and differences to an established data collection process (Renzella et al: Validity of a brief dietary survey to assess food intake and adherence to national dietary guidelines among Sri Lankan adults, submitted).

After participants reviewed the study information sheet and provided written informed consent for a recorded interview to take place, one female Sri Lankan researcher (BK) collected demographic data (age, ethnicity, gender, place of residence) and conducted semi-structured face-to-face interviews with each participant in their home. BK is an experienced data collector, interviewer, and researcher who has previously worked on health-related projects in Sri Lanka. Interviews were conducted in participants' preferred language: Sinhalese (*n* = 84) or English (*n* = 9). Functional equivalence of these data was achieved through verbatim transcription into the language used by the participant and free translation into English (*n* = 84) by a second female Sri Lankan researcher (SF). Ethics approval for this study was received from the University of Colombo and the University of Oxford. Participants were not incentivised or compensated for participation.

No time limit was set for interviews, which ranged from 15 to 30 min in duration. Participants were asked the following questions about responsibility for household food labour and differences in food choices and consumption patterns between household members:

1. *How do food* ***purchasing*** *responsibilities differ between household members?*
2. *How do food* ***payment*** *responsibilities differ between household members?*
3. *How do food* ***preparation*** *responsibilities differ between household members?*
4. *How do* ***cooking*** *responsibilities differ between household members?*
5. *How do food* ***choices*** *differ between household members?*
6. *How does food* ***consumption*** *differ between household members?*
7. *Is there anything you would like to tell me that I haven’t asked you about?*

### Data analysis

Transcribed and translated interview data were manually analysed following Braun and Clarke’s (2006) multiple step thematic analysis strategy [17]. Transcripts were first coded by question and then by participant. This allowed us to compare experiences between and understand dynamics within households. The first author (JR) read twice and then coded ten randomly selected transcripts covering the three districts on the basis of salient experiences that arose from the text. The codes and thematic groupings generated from this subset were discussed and revised with SF and NT before coding the remaining transcripts. Once all transcripts were coded, the wider research team discussed and revised thematic groupings (themes and subthemes). We were interested in all patterns and differences reported but expected most differences in food labour roles and responsibilities to be gender-based [12, 13]. We therefore deductively coded for gender differences in anticipation of confirming or challenging Sri Lankan gender norms. All other codes were generated inductively. To improve the internal generalisability of findings in this large sample size [18], we used quasi-statistics (percentage of participants who spoke to theme or subtheme) to give non-precise counts (*“some”, “few”, “many”)* an element of precision [19].

## Results

### Participant characteristics

Of the 94 people invited to participate in this study, 93 agreed to participate and were included in analysis. Participants ranged in age from 18 to 65 years old *(mean = 40.7, SD = 12.6)* and resided in urban (72%) or rural (28%) sectors in Colombo (60%), Kalutara (31%), and Trincomalee (9%) districts. Sixty-six percent identified as female and 34% as male. Four individuals (one woman and three men) reported living alone. All other participants lived in mixed-gender households. Participants self-defined their ethnicity as Sinhalese (86%), Tamil (8%) or Muslim (6%).

### Interview findings

Three key themes emerged from analysis of the interview transcripts. These themes and their associated subthemes (Table 1) are discussed in detail below.

**Table 1:** Interview findings: themes and subthemes (*N* = 93)

Themes	Subthemes
**1. Rigid gender differences exist in household food labour responsibilities**	*Women do most of the food-related work*
	*When labour is shared, roles are often gendered*
	*The “breadwinner(s)” pay for household food*
**2. There is some overlap between responsibility for food labour and influence over household diet**	*In many households, food decisions are driven by children, elderly family members, and individuals with medical conditions*
	*Influence over household diet is often gendered*
	*In households where all food preferences are appeased, women prepare multiple meals*
**3. The presence of intra-household consumption differences based on age and gender is uncommon, and its impact unclear**	*Traditional age and gender consumption hierarchies are not a feature of most households*
	*Where present, consumption hierarchies do not always result in unequal intra-household food distribution*
	*Women express a preference for ‘healthy eating’*

#### 1. Rigid gender differences exist in household food labour responsibilities

All respondents spoke to the topic of responsibility for household food purchasing, payment, preparation, and cooking through a gender lens. Where gender-based differences were reported, they appeared both clear-cut and set in stone. For example, with the exception of flexibility under extenuating circumstances such as illness, travel, or pregnancy, daily food roles and responsibilities were often categorised as belonging solely to the realm of women or men. The rigidity of these gendered roles and responsibilities is evidenced through reference to their repetitive (for example, *“I always”*) and axiomatic nature: *“My wife and three daughters do all the cooking. Males don’t participate at all. It is not our tradition” (Male, Colombo)*. With few exceptions, even in cases where responsibilities were shared between women and men, rigidity was a feature of these arrangements. For example, couples *always* shopped together or were responsible for each purchasing *specific* groceries.

##### Women do most of the food-related work

The responsibility for day-to-day food-related household labour is not shared equally between female and male household members. Cooking for the family, and particularly for children and the elderly, tends to be exclusively the responsibility of women. Compared with food preparation, where men assisted *“sometimes”*, *“very rarely”, “when my wife is indisposed”* or with *“specific tasks”*, cooking was most often described as being done *“mostly”*, *“exclusively”*, *“all”*, or *“100%”* by women.

*“I have never cooked in my life. My wife and my sister who is living with us do one hundred percent of the cooking.” (Male, Trincomalee)*

*“I do all of the food preparation. Actually, it doesn’t take me much time to cook. I like to manage the cooking and preparation of food without help or interference.” (Female, Colombo)*

*“I would say that 98% of the cooking is done by my wife. I have only cooked whilst I was in university. That too just rice, dhal curry and pol sambol.” (Male, Trincomalee)*

In two cases, cooking was a shared responsibility between a female and male family member.

*“My husband and I share this workload. My husband is the one who cooks the rice every day. He helps in scraping coconut, peeling onion, cleaning and cutting vegetables daily.” (Female, Colombo)*

Whereas food preparation and cooking were described as *“the type of work done by women”*, responsibility for food purchasing was more equally shared between women and men both across and within households. The majority of participants (67%) reported a gender difference in food purchasing responsibility, which was considered the sole responsibility of women in 48% and men in 44% of these households.

*“My husband is practically ignorant about food, food quality, what is needed or not needed. He has no interest or knowledge about how the rest of the family eats or about their food preferences. So, I am aware of everybody’s needs and I go out and do the purchases.” (Female, Trincomalee)*

*“My husband does the food purchasing. I rarely buy food items. If I do buy my husband will certainly find fault with what I buy.” (Female, Colombo)*

Shared responsibility for food purchasing reflected the experience of 25% of participants where women and men shopped for food together (during the same outing).

*“My husband and I both go together to shop for the food for the whole month. The vegetables of course are bought from time to time throughout the month.” (Female, Colombo)*

Some adults living with older family members reported that responsibility for food purchasing, whether shared between genders or assumed by one individual, fell to older family members.

*“My mother and father do this as a shared responsibility. In my house, I live with my mother and father.” (Male, Colombo)*

*“It is my elder sister or my brother in law who go and purchase the food.” (Male, Kalutara)*

##### When labour is shared, roles are often gendered

In households where purchasing and preparation responsibilities were shared between women and men, roles were often gendered based on their labour-intensiveness or the category of food (for example, fresh versus packaged) being purchased. Male assistance with food preparation was commonly described as *“rare”* or *“specific”.* In these cases, men were typically assigned more physically demanding tasks such as dehusking coconuts or cleaning and cutting fish and meat.

*“Usually it is my mother or my aunt that does the food preparation. But if there is a need to husk a coconut, clean a fish or cut a chicken, my father or I might help.” (Male, Kalutara)*

Gendered roles also extended to couples that grocery shopped together. When reported, differences were based on the type of food being purchased; women were responsible for purchasing fresh fruit and vegetables whereas men’s roles differed between households but often involved the purchasing of pre-prepared or ‘Western’ foodstuffs.

*“The cooked food or takeaway food is purchased by myself. My wife purchases the raw foods, rations etc.” (Male, Colombo)*

##### The “breadwinner(s)” pay for household food

It was rare that household food payments were exclusively the responsibility of women (5%). In some households, the responsibility was shared between women and men, but the most common response was that food payment was exclusively men’s responsibility (76%).

*“My husband is the only breadwinner. He pays for everything.” (Female, Colombo)*

*“The payment is done by my daughter as she is the only person who is earning sufficiently to buy food.” (Female, Colombo)*

Nineteen percent of respondents lived in households where payment responsibility was shared between women and men who all earned sufficiently to contribute. How much each person contributed (as a total amount or percentage contribution) was not disclosed by any of the study participants.

*“The responsibility is shared between men and women. My sister who lives in the household also earns. We share the responsibility. There is no clear demarcation about how much a man would spend or a woman would spend.” (Male, Colombo)*

#### 2. There is some overlap between responsibility for food labour and influence over household diet

The answer to *‘Who cooks?’* – women – was near ubiquitous across households, whereas responses to *‘What to cook and whose preferences to prioritise?’* varied from household to household. These decisions were less often influenced by the preferences of the individuals responsible for cooking and more often determined by the age, gender, health-status, and preferences of household members.

##### In many households, food decisions are driven by children, elderly family members, and individuals with medical conditions

In 30 % of households, the fussiness, appetites or vulnerable state of children and older family members – factors that have *“nothing to do with their gender”* – were described as being highly influential in determining what meals were consumed by the entire family.

*“The food choices we make are largely decided by what my son wants to eat. Almost all of the meals are planned and cooked according to his wish.” (Female, Kalutara)*

*“Predominantly my father’s choices are taken into account. That is not so much because he is a man. He is very old and he has low appetite. Therefore, we cook the foods that he would like to eat because he is old and feeble.” (Female, Colombo)*

Where one family member had a food allergy or was following a health-promoting/disease-preventing diet, the household menu was tailored accordingly, and all members would *“eat as they do”.*

*“The bulk of the food choices are tailored to suit my mother’s high cholesterol and diabetes. We all eat the food that suits her disease.” (Female, Colombo)*

*“As my husband is allergic to egg, we are very careful when using egg for cooking.” (Female, Trincomalee)*

##### Influence over household diet is often gendered

Twenty-five percent of respondents said that food choices were “*completely dominated”* by a male family member, whereas twenty-one reported that the female family member responsible for cooking made all of the food-related decisions. In both cases, the preferences of these individuals impacted the food intake of all other family members to varying degrees of contentment and implications for health.

*“It is always what my husband likes that gets cooked. There are many food items that he prefers that my daughter does not eat. But still the preference is given to what my husband likes.” (Female, Kalutara)*

*“My wife decides the food choices, what is bought and what is cooked. I never interfere in those choices. I eat to live, not live to eat. I’m very easy going about what is cooked and given to me.” (Male, Colombo)*

*The women in my family want to eat healthy. The men in my family prefer more unhealthy food. But invariably it is the decision of the men that will prevail. In Sri Lanka, typically men make decisions about everything. They are the head of the family. They also decide what and what not to cook. (Male, Trincomalee)*

##### In households where all food preferences are appeased, women prepare multiple meals

Some women tried to appease everyone’s food preferences simultaneously – whether based on individual tastes or dietary requirements – by cooking multiple dishes each meal. In every case, the additional effort required to satisfy and *“respect”* all preferences was conveyed. One respondent described this situation as the *“very hard daily balancing act”* that his mother has to perform because there is *“a huge issue of food choices in our house” (Male, Trincomalee).*

*“Well, my wife makes all the food choices and meal planning by herself. But the preferences of each person are taken into account. She will make many different dishes to cater to the different preferences. We have different preferences in the curries we like to eat. We don’t repeat the same dishes in the main meals.” (Male, Trincomalee)*

*“My husband is a vegetarian so I have to ensure that not even a cooking spoon is mixed with meat dishes when cooking his food. A lot of thought and energy goes into cooking his vegetarian dishes which are not shared by the other members of the family. I have to cook two sets of meals because my husband’s requirement is very specific.” (Female, Trincomalee)*

#### 3. The presence of intra-household consumption differences based on age and gender is uncommon, and its impact unclear

When asked how food consumption differs between household members, participants focussed on differences in the type, amount, and mode of food consumed between adult women, men, and children. The majority of households reported no age- or gender-based consumption differences or *“disparities”* in how food was distributed. Among those who reported consumption differences, no clear pattern emerged as to how these differences influenced or impacted the nutrition status of different household members.

##### Traditional age and gender consumption hierarchies are not a feature of most households

Seventy-two percent of participants did not indicate the presence of consumption hierarchies based on age or gender in their home: *“There is no gender discrimination in how the food is distributed” (Female, Kalutara).* One participant who distanced herself from the *“tradition”* of *“serving men first and most”* supplemented her answer with an explanation that consumption hierarchies of any kind are a tradition followed in *“poorer families”* where “*there is [not] enough food to go around” (Female, Colombo)*. Other respondents conveyed scenes of communal and egalitarian eating: *“We eat together. We eat as each of us please” (Female, Colombo).*

##### Where present, consumption hierarchies do not always result in unequal intra-household food distribution

Consumption hierarchies that saw men served first and more and/or women and men eating at different times were present in 28% of respondent households: *“First the food is served for my father. Then my mother serves the food to us, the children. My mother eats what is left after we all eat.” (Female, Kalutara)*. Notably, serving order did not always affect the amount of food available to others: “*My husband’s father is served first, but I would not say his gender affects the amounts eaten by anyone or the way it is eaten in my household*” *(Female, Kalutara).*

##### Women express a preference for ‘healthy eating’

Some participants (10%) noted gender-based differences between the types of food that women (*“healthy”* and *“fresh”*) and men (*“unhealthy”* and *“processed”*) prefer. When making these comparisons, participants described *“traditional foods”*, *“natural foods”*, and *“vegetables and fruit”* as healthy and *“fatty and processed food”*, *“junk food”*, *“cheese”*, *“sweets”*, *“carbohydrates”* and *“meat”* as unhealthy.

*“If my husband is making the food choices, he buys all the fatty and processed foods and sugary foods without even reading the labels. If I make the choices I focus more on dark green vegetables, leaves and fresh food.” (Female, Colombo)*

Gender differences in attitudes to healthy eating were less evident in children. Some parents said that their daughters took to their mother’s healthier preferences whereas others said they preferred *“much more meat like their father”.* Children, irrespective of gender, were generally described as having an appetite for *“oily and fried foods”*, *“short eats”*, and *“sweets”*.

*“My husband eats more carbohydrates and meats. My youngest daughter and I eat more vegetables and fruits” (Female, Colombo).*

*“My husband prefers more of processed food, cheese, and meats and so do my daughters. I prefer natural and traditional foods” (Female, Colombo).*

## Discussion

In recent decades, Sri Lanka has experienced rapid demographic, economic, nutrition, and social transitions [7, 20]. Our study shows that despite this dynamism, rigid gender differences in household food labour roles and responsibilities remain present. More in tune with this prevailing trend of change, however, is the finding that *“traditional”* consumption hierarchies with negative nutrition consequences are not a common feature of households in Colombo, Kalutara, and Trincomalee.

Among study participants, women do most of the food related work, and are almost exclusively responsible for cooking. This supports the literature that examines the role of women in Sri Lankan society and the labour force participation and success barriers they continue to face [12, 13, 21]. These studies, however, often aggregate domestic duties and childcare as a homogenous entity.

Moving beyond the question of ‘*who cooks?*’, our study findings add nuance to the narrative that all domestic chores are ‘women’s work’ by highlighting the role that men play. For example, whereas women are primarily responsible for food preparation and cooking, men are more involved in food purchasing and payment – the latter almost exclusively so. However, in almost all households, responsibility for food-related roles was inflexible. Without hesitation, participants were able to report who does what task and detail extenuating circumstances that might disrupt this arrangement. Even when responsibilities were shared, women and men assumed specific roles. For example, when food purchasing tasks were shared, women assumed responsibility for purchasing fresh and ‘healthy’ food whilst men shopped for pre-prepared or ‘unhealthy’ foods. An ethnographic study of ‘cooking life’ in Sri Lanka similarly discerns clear gendered trends in food procurement roles, which the author terms ‘structures of repetition’ [22]. In line with our study findings, these structures are described as being sometimes flexible but not often broken.

For many families, food decisions were driven by children, elderly family members, and individuals with medical conditions. Where this was not the case, influence over household diet was often gendered: either the women responsible for cooking made all of the food-related decisions or men’s preferences were prioritised. In almost all cases, the dietary requirements or preferences of these ‘food influencers’ impacted the diet of all household members. The exception to this trend was found in households where women appeased the conflicting preferences of all family members. Importantly, participant experiences confirm that responsibility for food labour is not always a proxy for diet decision-making power. Where gendered preferences for healthy eating were expressed, women tended to prefer *“healthy”*, *“fresh”*, and *“traditional”* foods whereas men were described as preferring *“unhealthy”*, *“processed”* products and *“more meat”*. Women’s higher awareness and knowledge of nutrition compared to men, who through their own admission had much less to do with food and in particular cooking, may in part explain this variation. Preference differences along health lines were often resolved by designing the household food menu around men’s ‘unhealthier choices’ or by cooking various meals to satisfy everyone. At a household level, the literature paints a mixed picture of the role of Sri Lankan women in decision-making [13, 23]. Our findings give a similarly heterogenous account. Specifically, the role that children’s fussiness and appetites play in influencing food decisions was a key and unexpected finding of this paper in a context where obedience towards parents is considered a core virtue [13]. Recent research in Taiwan and Singapore has also found that children exert considerable influence over their own and their family’s meal choices [24, 25]. In the latter study, Singaporean women noted that serving children foods that they liked was more practical and less wasteful. The accessibility and affordability of cooked meals purchased from food stalls made it relatively easy to satisfy children’s tastes, especially for working mothers [25]. To our knowledge, no Sri Lankan study has explored the role that either children or elderly family members and their associated health conditions play in family food choices and dietary health outcomes.

The presence of consumption hierarchies with associated negative nutrition consequences for women and children was not a feature of most study households, with some participants describing scenes of egalitarian and communal eating. Where present, many participants insisted that upholding this tradition of serving older males first did not impact other family members’ access to adequate nutrition. These findings are at odds with previous research. For example, the ethnographic study mentioned above identifies the presence of ‘homogeneous consumption groups’ that contribute to a lack of ‘table culture’ (i.e. where family members sit at the same table and share a meal together) [22]. Additionally, research conducted in urban Kandy reports the presence of both age- and gender-based intra-household calorie allocation biases [16]. The contrast between these findings and our own demonstrates the need for longitudinal mixed methods investigation into the context-specific presence, determinants, and outcomes of consumption hierarchies and intra-household food distribution both within and across South Asian countries.

### Strengths and limitations of this study

The main strengths of this study are twofold: the large sample size; and the qualitative and semi-structured method it followed, whereby participants were encouraged to share their experiences in their own time and words. This study has some limitations. Rich study findings may not be generalisable to the wider population and should be interpreted with caution. This is due to the qualitative methodology selected to meet exploratory study objectives and the limited geographic area from which participants were recruited. Interviews were conducted, translated, transcribed, and coded by female researchers. The gender of our data collection team may have impacted how questions were asked and the types of responses received. Without a comparison, it is difficult to gauge how data collection was impacted. A key limitation of this study is the absence of participant socio-economic and household composition data.

### Implications for policy and practice

This study adds to the growing body of literature that demonstrates the value of qualitative evidence for our understanding of the multiple factors that influence the appropriate and effective implementation of health policies and interventions [26]. Specifically, our study findings have implications for the improvement of efforts to promote healthy eating through the provision of healthy diet advice, guidance, and encouragement. The SLFBDGs are one such example. Among study participants, women were more likely to have the necessary knowledge and skills required to implement healthy diet guidance, but they did not necessarily have the decision-making power to change household dietary behaviours. Health promotion interventions that are blind to this disparity risk overemphasising the role of women in effective behaviour change and may inadvertently reinforce gender stereotypes or social conservatism that promotes the idea that a woman’s role is in the kitchen cooking for her family [27, 28]. Diet improvement efforts should therefore be directed to all family members – especially husbands and children whose attitudes and preferences are a crucial determinant of what and how family members eat. Given men are more involved in food payment and purchasing than any other aspect of food labour, practical healthy purchasing advice to support various point-of-sale nutrition interventions could be included in dietary guideline documents that aim to ‘provide the general public with solutions that they can easily follow’ [9]. Equally, all family members are involved in the consumption of food. Information to support the creation of healthy consumption environments, for example the promotion of family meals or inclusive ‘table culture’, could improve healthy eating behaviours in children, adolescents, and adults [29–31]. Precedent for this can be found in the Brazilian, Korean, and Mediterranean diet guidelines, to name a few, where convivial consumption environments and the practice of enjoying meals in the company of others are core recommendations [32–34]. For many women in this study, balancing the food choices of family members was a difficult task and it was often people’s tastes rather than health considerations that determined meals served. Dietary guidance that accounts for taste alongside health considerations may therefore be more useful to women who find themselves in this difficult decision-making role.

## Conclusion

This study shows that household gender and age dynamics in Colombo, Kalutara, and Trincomalee influence what and how people eat. Whilst confirming that women are disproportionately involved in food preparation and cooking, this study also contributes new and important findings about diet decision-making and the presence and potential impacts of intra-household consumption differences. Dietary interventions with the aim of effectively and appropriately influencing day-to-day practice should therefore be developed with a contextualised awareness of who is responsible for, who is able to perform, and who influences targeted behaviours. The use of qualitative research can help generate such information.

## Supplementary information

**Additional file 1.**

## Data Availability

The data generated and analysed during the current study are not publicly available as ethical approval does not extend to the sharing of participant data or interview transcripts beyond study authors.

## Abbreviations

DBM: Double Burden of Malnutrition
GND: Grama Niladhari Division
NCD: Noncommunicable disease
SLFBDG: Sri Lankan food-based dietary guidelines
WHO: World Health Organization
