# Supplementary material for: Food labour, consumption hierarchies, and diet decision-making in Sri Lankan households: a qualitative study
Source: BMC Nutr. 2020 Nov 20;6:64. doi: 10.1186/s40795-020-00389-w (PMC7678094; doi:10.1186/s40795-020-00389-w)
Supplement: Supplementary file 1 — Additional file 1. [file 40795_2020_389_MOESM1_ESM.docx]

The following interview questions were developed for this study:

1. *How do food* ***purchasing*** *responsibilities differ between household members?*
2. *How do food* ***payment*** *responsibilities differ between household members?*
3. *How do food* ***preparation*** *responsibilities differ between household members?*
4. *How do* ***cooking*** *responsibilities differ between household members?*
5. *How do food* ***choices*** *differ between household members?*
6. *How does food* ***consumption*** *differ between household members?*
7. *Is there anything you would like to tell me that I haven’t asked you about?*
